# Data-Quality Assessment Signals Toxic-Site Safety Threats and Environmental Injustices

**DOI:** 10.3390/ijerph18042012

**Published:** 2021-02-19

**Authors:** Kristin Shrader-Frechette, Andrew M. Biondo

**Affiliations:** 1 Department of Biological Sciences, 100 Malloy Hall, University of Notre Dame, Notre Dame, IN 46556, USA; 2 Department of Economics, 3060 Jenkins Nanovic Hall, University of Notre Dame, Notre Dame, IN 46556, USA; abiondo@nd.edu

**Keywords:** CBRE/Trammell Crow, data-quality analysis, environmental justice, hazardous waste, pollution, toxin, trichloroethylene (TCE), vapor intrusion, volatile organic compound

## Abstract

Most hazardous-waste sites are located in urban areas populated by disproportionate numbers of children, minorities, and poor people who, as a result, face more severe pollution threats and environmental-health inequalities. Partly to address this harm, in 2017 the United Nations unanimously endorsed the New Urban Agenda, which includes redeveloping urban-infill-toxic-waste sites. However, no systematic, independent analyses assess the public-health adequacy of such hazardous-facility redevelopments. Our *objective* is to provide a preliminary data-quality assessment (PDQA) of urban-infill-toxic-site testing, conducted by private redevelopers, including whether it adequately addresses pollution threats. To this end, we used two qualitative, weight-of-evidence *methods*. Method 1 employs nine criteria to select assessments for PDQA and help control for confounders. To conduct PDQA, Method 2 uses three US Environmental Protection Agency standards—the temporal, geographical, and technological representativeness of sampling. Our Method 1 *results* reveal four current toxic-site assessments (by CBRE/Trammell Crow, the world’s largest commercial developer); at all of these sites the main risk drivers are solvents, volatile organic compounds, including trichloroethylene. Our Method 2 *results* indicate that all four assessments violate most PDQA standards and systematically underestimate health risk. These results reveal environmental injustice, disproportionate health threats to children/minorities/poor people at all four sites. Although preliminary, our *conclusion* is that alleviating harm and environmental-health inequalities posed by urban-infill-toxic-site pollution may require improving both the testing/cleanup/redevelopment requirements of the New Urban Agenda and the regulatory oversight of assessment and remediation performed by private redevelopers.

## 1. Introduction

Children, minorities, and poor people are disproportionately represented among those living near hazardous-waste sites; as a result, they face at least five times higher toxic exposures and environmental-health inequalities than other citizens [1]. They bear disproportionate rates of birth defects, e.g., [2], cancers, and other harm; children are threatened most [3]. 

In poor- and middle-income nations, at least 200 million people are at risk from thousands of mostly urban hazardous-waste sites [4], only 3% of which have begun any risk assessment or mitigation [5]. In the United States, more than 120,000 toxic sites await adequate cleanup, and thousands of already-closed, once-considered-clean locations are being re-evaluated because of health threats [6].

### 1.1. The New Urban Agenda

Globally these mostly urban, toxic-waste sites will soon threaten even more people because, by the year 2050, 70% of the world’s population will live in cities, up from the current 55% [7]. To solve the problems of unremediated hazardous-waste sites and increased urbanization, sprawl, and concentrated city pollution, in 2017 the United Nations (UN) General Assembly unanimously endorsed the New Urban Agenda [8]. All member nations pledged to accomplish urban upgrading, sustainability, and population densification, partly through urban-infill redevelopment [8], construction on land that needs rehabilitation, such as hazardous-site remediation. 

To promote urban-infill-toxic-site redevelopment, the World Bank [9,10], the European Commission, and the Urban Agenda Partnership of the EU [11,12] appear to be encouraging private-sector remediation of hazardous sites through tax breaks and removing private-sector liability. Both the World Bank and the EU thus appear to be encouraging nations to follow the urban-infill-toxic-site redevelopment model of the United States. In the US, most toxic-site testing/remediation is semi-privatized or “voluntary,” conducted by private parties, usually commercial redevelopers of the hazardous sites, under varying degrees of government oversight [13]. The private parties negotiate levels of assessment/cleanup on state (not federal) hazardous sites and, in exchange, receive government-liability protection plus regulatory, financial, and other benefits [14].

### 1.2. Objective and Hypothesis

Despite the UN’s New Urban Agenda and the World Bank’s and EU’s apparent promotion of semi-privatized cleanup, information about the public-health consequences of semi-privatized toxic-site cleanups is largely anecdotal. On one hand, semi-privatized assessments/cleanups are cheaper and faster but, on the other hand, critics charge that semi-private testing/remediation often fails government scientific-data audits, is “typically driven by purely [private] financial motives” [15], and frequently has inadequate government oversight, e.g., [16]. In 2019, two economists tried to sort out these competing stances; they claimed to provide the first analysis of the public costs and benefits of privatized toxic-site assessment/cleanup but assessed only remediation-related, property-value increases and ignored all public-health risks/costs/benefits [13] (p. 369). This analysis begins to remedy this public-health data gap.

No one has conducted an independent, systematic, proactive analysis of whether semi-privatized urban-infill-toxic-site testing/remediation actually satisfies scientific data-quality-assessment (DQA) standards, a necessary condition for protecting public health and environmental justice/equality. The objective of this analysis is to conduct a preliminary investigation (see the Section 2), a preliminary DQA (PDQA) of possible public-health threats from semi-privatized hazardous-site assessments/cleanups. Our specific hypothesis is that interested parties’ testing of urban-infill hazardous-waste sites contaminated with volatile organic compounds (VOCs) may be non-representative, fail PDQA, and fail to adequately assess carcinogenic vapor intrusion (VI), that is, into-building migration of subsurface, gaseous VOCs [17]; therefore such testing may fail to address pollution threats and environmental injustice/inequality that the New Urban Agenda was designed to correct.

Perhaps one reason no one has yet conducted an independent analysis of the quality of vapor-intrusion studies of toxic sites is that VI assessment is relatively new. In the 1960s, scientists recognized radon and petroleum-landfill VI, but not until the 1970s did they understand that VOCs are also VI-inhalation carcinogens. Similarly, not until the late 1980s did the US Environmental Protection Agency (EPA) warnings to assess VOC VI; it did so in its Risk Assessment Guidance for Superfund sites and its draft VI Guidance [17].

In assessing toxic-site VOC testing, we follow US EPA and define representativeness in terms of three standards: the “temporal, geographical, and technological correlation” or representativeness between sampling data and the true population of interest [18] (p. 11). We investigate representativeness because it is a key indicator of sampling/testing quality, arguably the most “critical component” of DQA, the main point of which is to ensure that testing captures the population of interest [18] (pp. 3–4). 

### 1.3. Why Our Hypothesis Is Important

Testing our hypothesis (see above), that toxic-site sampling may fail PDQA and thus threaten public health and environmental justice, is important partly because pollution is the largest environmental cause of global disease and premature death [19]. Even in developed nations such as the United States, 89% of toxic sites pose “serious health risks to communities,” partly because the percentage of health-threatening sites is increasing, not decreasing [20]. Yet flawed toxic-site testing or cleanup could allow such health harm to continue, e.g., [21], thus disproportionately affecting children, minorities, and poor people [19]. Not only is chemical production moving to low- and middle-income countries [19] but also, throughout the world, “more deprived populations tend to live closer to hazardous sites and to be more exposed to their emissions” [22].

## 2. Materials and Methods 

This PDQA of the representativeness of toxic-site-VOC sampling relies on two main observational, weight-of-evidence (WoE) methods. (WoE procedures are qualitative; they require assembling/weighting/evaluating different, site-specific lines of evidence in order to come to the most scientifically defensible conclusion [23], in this case, about potential violations of DQA standards.) 

The first method of our analysis selects toxic-site assessments for PDQA evaluation, and these assessments are the main materials used in our analysis. Our second method employs three main US EPA representativeness standards for evaluating soil-gas sampling through PDQA. These representativeness standards are found in additional materials used in our analysis, namely, US EPA’s guidance on DQA.

### 2.1. Method 1

To select current toxic-site assessments for PDQA evaluation, Method 1 employs nine criteria, listed in Table 1. They specify selecting only current, maximum-threat, toxic sites for testing evaluation that are being assessed through semi-privatized schemes and that have publicly accessible documents. Consistent with the UN’s New Urban Agenda and earlier paragraphs, these nine criteria specify including only sites that are slated for urban-infill-redevelopment (Criterion 1, Table 1); undergoing semi-privatized assessment/remediation (Criterion 2, Table 1); whose subsurface VOCs can cause vapor intrusion (VI) (Criterion 3, Table 1). 

Regarding Criterion 3 in Table 1, for 3 reasons, we investigated only toxic sites whose major contaminants include subsurface VOCs. *First*, although subsurface threats from VOCs perchloroethylene (PCE) and trichloroethylene (TCE) have been known at least since 1969, when disproportionate numbers of children in Woburn, Massachusetts began dying of leukemia [24], subsurface VOCs continue to cause groundwater and VI contamination, resulting in widespread cancers, birth defects, and other harm, e.g., [25]. *Second*, although global data are not available, the majority of US toxic sites, at least 88,000 [26], could cause public exposure to carcinogenic VI, as subsurface VOCs are within at least 100 ft of buildings [17]; other nations likely face similar VI threats. *Third*, despite these VOC public-health risks, and the ubiquity of urban sites contaminated with solvent VOCs, VI field investigations are common in only 5 countries: Australia, Canada, Denmark, the United Kingdom, and the United States [27].

Regarding Criterion 4 in Table 1, to help avoid the confounder of accidental or ignorance-based violations of representative-sampling standards, this criterion specifies evaluating only sites whose testing was performed by/for the world’s largest commercial developer, CBRE/Trammell Crow Company (CBRE/TCC), the presumed trendsetter for other toxic-site redevelopers. That is, because CBRE/TCC is “the industry leader in brownfields development” [28], who says it “pioneered the concept of Privatized Remediation” [29], evaluating CBRE/TCC testing should provide a conservative, best-case estimate of industry toxic-sampling practices. 

Regarding Criterion 5, to help avoid the confounder of inadequate law/regulation/guidance causing flawed testing, this criterion specifies evaluating only California hazardous-site sampling. Why? California toxic-site laws/regulations generally are stricter/more comprehensive than federal laws [30], and, along with New Jersey, California has the highest number of the deadliest US toxic-waste sites, Superfund/Comprehensive Environmental Response, Compensation, and Liability Act (CERCLA) facilities [31].

To operationalize Method 1′s preceding assessment-selection criteria 4–5 in Table 1, we selected only toxic-site assessments listed in at least one of three sources. These sources are (1) CBRE/TCC’s Environmental Asset Services, Inc. Division projects, including its Brownfields Acquisition and Development projects [28]; (2) CBRE/TCC’s projects overseen by Robert Chute, Senior Vice President for environmental risk management [32]; and (3) “Trammell Crow Residential” projects listed in the state (California Environmental Quality Act) CEQAnet database [33].

Criterion 6 in Table 1 requires evaluating only assessments that include soil-gas sampling, both because VOCs usually first contaminate soil-gas/soil, then travel to groundwater/indoor air, and because soil gas, not soil, provides more accurate VOC measurements [17]; see [34]. Criterion 7 requires evaluating only post-2011 assessments, as 2011 is the issue date of the dominant, still-current, government soil-gas-testing guidance [17]. 

Criterion 8 requires that assessments to be evaluated have publicly accessible soil-gas data sheets and other documents on the California Department of Toxic Substances Control (DTSC) Envirostor website [35]. This analysis relies only on publicly accessible data.

In order to select especially health-threatening hazardous-waste locations, Criterion 9 of Table 1 requires evaluating only sites that include trichloroethylene (TCE) contamination. TCE is a no-safe-dose, genotoxic carcinogen that is up to 10 times more harmful for children than adults [36]. Because even a single, brief, 0.5 µg/m^3^ TCE airborne exposure can “produce an adverse developmental effect” on the blood, kidneys, and liver and on the cardiovascular, immune, nervous, and respiratory systems [34,37] (p. 2), in 2014 the US government mandated very strict TCE public-health-protection levels [34]. 

### 2.2. Method 2

Method 2 employs three US EPA representativeness standards for evaluating soil-gas sampling (Table 2), namely, “temporal, geographical, and technological representativeness,” as defined earlier [18] (p.11). Method 2 operationalizes/applies these three standards through 10 representativeness questions (RepQ), based on California DTSC soil-gas-testing “requirements” [17]. 

*The first of 3 main representativeness standards, temporal representativeness*, measures the correlation between the date of sampling and the year the study represents; US EPA says testing data must be less than 3 years old but, to “guarantee” temporal representativeness, from only the last year [18]. Therefore, to assess temporal representativeness, Method 2 asks

- (RepQ1) Are samples less than 3 years, preferably within 1 year, old?

*The second representativeness standard, geographical representativeness*, measures whether data collection is in “the desired geographical location” [18], that is, whether soil-gas samples follow government “requirements” (i) to be “collected near contaminant sources” [17]; (ii) to follow the Soil-Gas Advisory (SGA) mandate to trace all contaminant plumes to their 3-D termination [38]; (iii) to follow the SGA mandate to provide a 3-D, subsurface-contamination delineation [38]; (iv) to follow the SGA mandate to conduct sampling dense enough to provide all-contaminant, isoconcentration-contour maps [17]; and (v) to follow the SGA mandate to ensure “a minimum” of two sub-slab sample locations per building [38]. Following the preceding 5 soil-gas-testing “requirements”, Method 2 assesses geographical representativeness by asking, at all sites assessed, e.g., [39,40,41,42,43,44]:

- (RepQ2) Were data collected near contaminant sources?
- (RepQ3) Do samples trace the full horizontal/vertical extent of vapor-con-taminant plumes?
- (RepQ4) Do samples provide a 3-D delineation of all subsurface contamnants?
- (RepQ5) Are data dense enough to provide isoconcentration-contour maps for all toxins?
- (RepQ6) Do data provide at least two sub-slab-sampling locations per building?

*The third representativeness standard, technological representativeness*, measures whether the scope of sampling technology adequately captures at least 4 aspects of the data source or target system: (i) the *design conditions* imposed on sampling; (ii) the *material stability* (input/output) of sampling products; (iii) the *process scale*, sampling output per time/space; and (iv) the site-specific *operating conditions* [45]. Government regulatory guidance captures (i)–(iv) by means of “requirements” for soil-gas sampling, specifying that reporting/detection limits must be sensitive enough for a “screening evaluation”, must use permanent/semi-permanent soil-gas wells (to determine same-location, same-contaminant levels through time, thus whether pollutants are migrating), must show seasonal/temporal contaminant variations, and must be conducted under “steady-state conditions” [17]. Following the preceding government sampling requirements, Method 2 assesses technological representativeness by asking:

- (RepQ7) Are method-reporting limits sensitive enough for a screening evaluation?
- (RepQ8) Are samples from permanent/semi-permanent soil-gas wells?
- (RepQ9) Does soil-gas sampling output show seasonal/temporal varia-tions?
- (RepQ10) Is soil-gas sampling conducted under “steady-state conditions”?

## 3. Results

### 3.1. Method 1

Four Los Angeles County hazardous-waste sites meet our preceding 9 criteria for selecting high-risk locations. Because the developers claim they have completed and submitted sampling at all four locations assessed in this study, all testing results are supposedly final at each of these four sites. These are (1) the 9-acre former US Naval Ordnance Testing Station, Pasadena, California (NOTSPA), Envirostor ID 19970020 envirostor.dtsc.ca.gov/public/profile_report?global_id=19970020; (2) the 10-acre heavy-manufacturing site, Monrovia, California, Envirostor ID 60002828 envirostor.dtsc.ca.gov/public/profile_report?global_id=60002828 and cityofmonrovia.org/home/showdocument?id=21816; (3) the 51-acre former Raytheon Missile site, in California’s Canoga Park neighborhood, Envirostor ID 80001366 envirostor.dtsc.ca.gov/public/profile_report?global_id=80001366; and (4) the 18-acre former Santa Fe Railyards, Boyle Heights, California, Envirostor ID 19400008 envirostor.dtsc.ca.gov/public/profile_report?global_id=19400008.

### 3.2. Method 2

To evaluate the four soil-gas assessments revealed by Method 1, we used 10 representativeness questions (RepQ) based on US EPA DQA standards and California DTSC soil-gas “requirements” (see Section 3.1; see the authors’ Table 2). These 10 Method 1 questions include 1 question for temporal, 5 for geographical, and 4 for technological representativeness.

#### 3.2.1. Temporal Representativeness (RepQ1): Outdated Sampling

Temporal representativeness is important because only recent sampling ensures that site toxins have not migrated either deeper or further offsite, thus threatening additional victims. Dated sampling can reveal only some of the locations of toxic waste, as it might not capture later contaminant migration. 

At Pasadena’s former NOTSPA, a US military weapons-testing site, the latest soil-gas sampling was in 2007 (Table 3), submitted late in 2017 for approval in 2019 [39]. Thus, this sampling is outdated by 7–9 years because it was not conducted either within the last 3 years or, preferably, within the year before submission. The 2011 Monrovia site samples, submitted for approval in 2020 [40], are outdated by 6–8 years. The 2012 Canoga Park site samples [41], submitted for groundwater-closure approval in 2017 [42], are outdated by at least 2–4 years. The 2017 Boyle Heights samples, submitted in 2019 for site closure [43], were submitted within 3 years, but not within 1 year, of sampling. Thus, they are not outdated, yet they fail to “guarantee” US EPA temporal representativeness. Sampling data at three sites violate the temporal representativeness standard RepQ1, thus support our hypothesis of CBRE/TCC violations of government sampling requirements. Sampling data at one site satisfy RepQ1, and thus do not support our hypothesis.

#### 3.2.2. Geographical Representativeness (RepQ2): Near-Source Sampling

None of the soil-gas data for the four sites includes sampling near all sources, many of which remain unknown, because no assessments conducted a full-site, soil-gas survey, randomized sampling, or grid-based sampling. Consequently, Pasadena documents identified only 5- and 15-ft sources for only 2 (carbon tetrachloride and perchloroethylene) of roughly 35 contaminants [44]. Monrovia site documents identify no subsurface soil-gas-contaminant sources and include only 21 total, single-depth, soil-gas samples from one small area of the 10-acre site [40].

CBRE/TCC conducted no Canoga Park sitewide soil-gas survey, despite California DTSC’ repeatedly requesting it [42]; consequently, site documents list no soil-gas contaminant (secondary) sources. Similarly, Boyle Heights sources have not been (and will not be) removed, as regulators approved Boyle Heights site closure, despite the fact that half of site soil-gas wells (24 of 44) show currently increasing levels of VOCs such as PCE and TCE, and testing has never been done to locate VOC sources 100 ft above groundwater (Table 2 in [43]). In summary, sampling data at all four sites violate RepQ2, thus support our hypothesis of CBRE/TCC violations of government sampling requirements.

#### 3.2.3. Geographical Representativeness (RepQ3–5): All-Contaminant Perimeter Tracing

As indicated, no sites had full soil-gas surveys, randomized sampling, or grid-based sampling. As a result, no assessments trace all-contaminant extent (RepQ3), their 3-D contours (RepQ4), or all-contaminant, all-depth isoconcentration contours (RepQ5) for the Pasadena [44], Monrovia [40], Canoga Park [41], or Boyle Heights sites [43].

For instance, instead of following RepQ3, Pasadena assessors tested highly mobile soil gas only halfway down to groundwater; they stopped testing despite soil-gas concentrations, e.g., 20,600 µg/m^3^ carbon tetrachloride at location NMSD3, up to 307,500 times above their own screening/health-protective level (0.067 µg/m^3^) (Table 3 in [39]). Instead of following RepQ4, they tested no offsite-migration plumes, despite mobile, subsurface, site-boundary, contaminant concentrations as high as 298,000 times above health-protective/screening levels, e.g., 137,000 µg/m^3^ perchloroethylene at location V-9 (Table 3 in [39]). Instead of following RepQ5, they provided only 2-D isoconcentration maps for only 2 of 35 site contaminants, but only at 5 and 15 ft [44]. 

Likewise, because Monrovia assessors tested only shallow soil, only at one small area of the site, they failed to meet RepQ3, RepQ4, and RepQ5 [40]. Because Boyle Heights assessors tested soil gas within only two-thirds of the distance to groundwater, they too failed to meet RepQ3, RepQ4, and RepQ5 [43].

Instead of following RepQ3, Canoga Park assessors sampled only three soil-gas locations, each only 20% of the way down to groundwater [41] (Figure 4); see [42]). They then stopped downward testing despite trichlorofluoromethane (TCFM) concentrations (260,000 µg/m^3^ at location SG2011-1) 200 times above their own health-protective/screening level (1300 µg/m^3^) [39]; yet they claimed TCFM “concentrations do not pose a significant impact to the site or surrounding area” [41]. However, because TCFM groundwater-well concentrations (under/slightly downgradient from all excess soil-gas locations, shown in the sampling-location map) have been increasing (in well CM-10 since 2004 and in MW-31 since 2006) Figure 3 in [41]. TCFM obviously is migrating to groundwater and perhaps offsite. Instead of following RepQ5, Canoga Park assessors provided an “approximate” and “postulated” 2-D isoconcentration map, but only for 5–6-ft depth and only for TCFM, based only on a total of three, one-time, temporary-well samples (Figure 4 in [41]). In summary, because sampling data violate RepQ3–5 at all four sites, these results also support our hypothesis of CBRE/TCC failure to follow government sampling requirements.

#### 3.2.4. Geographical Representativeness (RepQ6): Sub-Slab Sampling

Because of VOC volatilization at open-air-sampling spots and especially shallow, sandy soil at all four sites [40,41,43,44], only building sub-slab sampling is representative of the highest levels of contaminants that could cause carcinogenic vapor intrusion [38]. Nevertheless, neither Pasadena [39], Monrovia [40], Canoga Park [41], nor Boyle Heights [43] assessors provided the required two-location, sub-slab samples for each site building (Table 4). Pasadena assessors, for example, collected no sub-slab samples for 89% of 26 currently occupied buildings covering two-thirds of the site [44]. Monrovia assessors conducted sub-slab sampling for only four of seven buildings [40] (Figure 3). Canoga Park assessors conducted no sub-slab sampling (Figure 4 and Table 1 in [41]). In summary, sampling data violate RepQ6 at all four sites and support our hypothesis of CBRE/TCC violations of government sampling requirements.

#### 3.2.5. Technological Representativeness (RepQ7): Sensitive-Detection Limits

None of the four sites satisfies the technological-representativeness standard of having method-reporting/detection limits at least as sensitive as screening/health-protective levels (Table 4). 

For all site contaminants, CBRE/TCC’s Pasadena reporting/detection limits are 20 µg/m^3^, up to three orders of magnitude less protective than DTSC’s required health-protective/screening levels, e.g., 0.067 µg/m^3^ for carbon tetrachloride [39]. CBRE/TCC’s Monrovia-site, 2020 reporting/detection limits, are 20 µg/m^3^ [40], up to two orders of magnitude less protective than DTSC’s required health-protective/screening levels (e.g., 0.46 µg/m^3^ for perchloroethylene, 0.48 µg/m^3^ for trichloroethylene) [39]. CBRE/TCC’s Canoga Park reporting/detection limits (e.g., 3.4 µg/m^3^ for perchloroethylene) [41], in documents submitted in 2017 [42], are up to 10 times less protective than DTSC’s required health-protective/screening levels, e.g., 0.46 µg/m^3^ for perchloroethylene [39]. CBRE/TCC’s Boyle Heights reporting/detection limits, e.g., 100 µg/m^3^ for perchloroethylene and trichloroethylene [43], are up to three orders of magnitude less protective than DTSC’s required health-protective/screening levels, e.g., 0.46 µg/m^3^ for perchloroethylene and 0.48 µg/m^3^ for trichloroethylene [39]. In short, sampling data violate RepQ7 at all four sites and thus support our hypothesis of CBRE/TCC violations of government sampling requirements.

#### 3.2.6. Technological Representativeness (RepQ8–9): Seasonal Semi-Permanent-Well Sampling

None of the four sites satisfies the technological representativeness standards of employing only permanent/semi-permanent wells (RepQ8) and measuring sitewide, same-location, same-contaminant seasonal/temporal variations (RepQ9) (Table 5).

Given only temporary wells, the preceding table shows that neither Pasadena [39] (Table 3), nor Monrovia [40], nor Canoga Park [41] (Table 1, Figure 4) satisfies RepQ8–9. Because only 5% of soil-gas wells on the 18-acre Boyle Heights railyard toxic site are semi-permanent, they are likely to be non-representative of sitewide seasonal/temporal variations [43]. In summary, because sampling data violate RepQ8–9 at all four sites, these results also support our hypothesis of CBRE/TCC violations of government sampling requirements.

#### 3.2.7. Technological Representativeness (RepQ10): Steady-State Sampling

Because none of the four site assessments employed only permanent/semi-permanent wells for same-location, multi-season sampling, no testing was conducted under known, sitewide, steady-state conditions, thus violating RepQ10 (Table 6). Moreover, although Pasadena [39], Monrovia [40], and Canoga Park [41] employed only single-sample temporary wells, making steady-state assessment impossible, most soil-gas wells on these sites showed increasing-with-depth contaminant levels. Despite site closure, Boyle Heights testing showed increasing soil-gas contaminant levels over the previous two years, 2016–2017 [43] (Table 2). Consequently, none of the four sites provides evidence for steady-state-contaminant conditions that might confirm no potential for offsite and into-groundwater migration, thus additional public-health threats. In summary, sampling data violate RepQ10 at all four sites and thus also support our hypothesis.

In summary, the preceding results show that all four toxic sites violate sampling-representativeness standards RepQ2–10, three sites violate RepQ1, and one site satisfies RepQ1. Thus 39 of 40 testing results support our hypothesis of CBRE/TCC violations of government sampling requirements. However, 1 of 40 testing results does not support our hypothesis. 

## 4. Discussion

One *explanation for many of these 39 of 40 sampling-representativeness violations* might be purely judgmental sampling. That is, at all four sites, assessors chose sampling locations/types purely on the basis of their beliefs/experience, and not based on any systematic plan that would investigate contaminants across the entire site. None of the four locations had a waste-characterization, sitewide soil-gas survey, or grid or randomized sampling; all had only judgmental sampling. Yet as EPA’s [45] (p. 13) representative-sampling guidance notes, “randomization is necessary in order to make probability or confidence statements” about sampling results; judgmental-sampling approaches can establish only threat, but “a larger number of samples are needed to characterize wastes, and sampling locations should be selected using random, systematic-grid, and systematic-random-sampling techniques.” Because of failure to employ either grid-based or randomized sampling, probability or confidence guarantees about sampling are impossible at all four toxic sites. Obviously, however, adequate public-health protection arguably requires such guarantees, especially to avoid health threats that might result from redevelopers’ financial conflicts of interest. Otherwise, redevelopers could conduct purely judgmental sampling that serves their commercial interests more than public-health protection.

### 4.1. Public-Health Implications

One of the most significant aspects of these 39 sampling-representativeness violations is that all of them appear to cause risk underestimation, and none appears to cause risk overestimation. No site results revealed the highest/worst *magnitude of contamination* because no tests sampled all of the highest-contamination areas (see the authors’ Table 8). That is, the preceding results show that assessors failed to conduct all required near-source sampling (RepQ2), under-slab sampling (RepQ6), and long-term sampling (RepQ8), including sampling showing seasonal/temporal variations (RepQ9). Likewise, no results showed the greatest/highest *extent of contamination* because tests at most sites used outdated samples (violating RepQ1) that failed to account for contaminant migration, and they did not trace contaminant extent (RepQ3), 3-D-contaminant outlines (RepQ4), and isoconcentration contours (RepQ5) (see the authors’ Table 8). Results likewise show that assessors also employed insensitive tests (RepQ7) and conducted no tests under steady-state conditions (RepQ10), all of which failed to capture the highest levels of most site pollutants. Instead, this systematic contaminant underestimation, at all four sites, indicates that pollutants could be migrating offsite, into buildings (VI), or into groundwater and thus causing public-health harm. The fact that all 10 PDQA violations cause risk underestimation suggests that the results are systematically biased; normal or expected errors likely would cause a mix of over- and under-estimates.

Flawed sampling that causes such risk underestimates is especially worrisome because whenever toxic-site testing underestimates health risks, by failing to conduct complete, adequate sampling, the resulting hazardous-site cleanup is likely also to be incomplete; this is because toxic-chemical threats that are not detected, through testing, cannot be remediated. Yet if toxic-site health threats are not remediated, obviously people could be harmed.

Consider four specific public-health threats from the toxic sites because of flawed testing and resulting risk underestimation. *First*, because the Pasadena [44] and Monrovia [40] toxic sites are residential-apartment redevelopments where site groundwater has never been tested (see the authors’ Table 7), they could be massively contaminated. Yet together these apartments will house roughly 1000 families, including a significant portion of families in affordable-housing units. These apartments also will likely have disproportionate numbers of children; site plans show 40% of apartments have two/three bedrooms for families with children, whereas only 31% of California households include age-18-and-younger children [46]. As already noted, these children are especially vulnerable to site trichloroethylene because of potential VI [34,36].

*Second*, the Pasadena–Monrovia toxic-site-apartment-residence developments are the only of the four sites built immediately adjacent to a freeway; both sites abut a 10-lane, Los Angeles, East–West, diesel-truck artery [47], Interstate 210. Thus, both CBRE/TCC redevelopments violate the California Air Resources Board recommendation against building homes/medical facilities/daycare centers/schools/playgrounds within 500 ft of freeways [48].

Not only are residents of these two sites (Pasadena and Monrovia) likely to be disproportionately children, minorities, and poor people, as Section 4.1 and Section 4.1.2 reveal, but also their beside-the-freeway living will exacerbate whatever health problems they already have. Air-pollution exposure worsens mortality from already-existing cancer and thus worsens existing environmental inequities and injustice [49].

*Third*, our results likewise show that the public-health consequences of the preceding risk/contaminant underestimates could be especially dangerous at the Pasadena [44] and Canoga Park [41] sites. This is because both sites were US military installations (see Table 7), and both sites developed/tested/manufactured nuclear and other classified (secret) missiles/torpedoes.

#### 4.1.1. Sampling Misrepresentations Threaten Near-Site and Onsite Populations

A *fourth* public-health threat is that, despite the fact that the four site assessments together violate 39 of 40 DQA “requirements,” at every site CBRE/TCC misrepresented its non-representative sampling as safe (see the authors’ Table 8), in order to justify performing no additional remediation at Pasadena [50], Monrovia [51], Canoga Park [41], and Boyle Heights [43]. For instance, at Pasadena, CBRE/TCC wrote to the state regulator, city officials, and city residents saying that all site contaminants “were fully investigated” [50]. At Boyle Heights, CBRE/TCC again wrote to the regulator and citizens that “soil vapor contamination has been addressed…and no further action is warranted” [43]. Yet as the preceding results have already noted, 24 of 44 semi-permanent soil-gas wells showed increasing levels of trichloroethylene or perchloroethylene over the previous two years (Table 2) [43]. Some levels were as high as 130,000 µg/m^3^ perchloroethylene and 28,000 µg/m^3^ trichloroethylene, respectively, 282,600 and 58,300 times above required DTSC health-/screening levels (0.46 µg/m^3^ perchloroethylene; 0.48 µg/m^3^ trichloroethylene) [39]. Such increasing contaminant concentrations indicate an ongoing and increasing threat, almost certainly because site contaminants have not been removed.

CBRE/TCC’s 2018 Canoga Park misrepresentations are especially disturbing because its own documents repeatedly and explicitly contradict its written claims to state regulators and near-site residents. CBRE/TCC petitioned the regulator for no further cleanup action, on grounds that site TCFM (a) is not “a carcinogen and is low in toxicity”, (b) is “below all available Environmental Screening Levels (ESLs)”, and (c) exhibits “no significant increase of concentrations with depth” [41]. However, claims (a)–(c) are false. 

*First*, CBRE/TCC’s 2018 low-toxicity claim is false because TCFM is an ozone-depleting and global-warming chemical, which has been banned internationally since the 1986 Montreal Protocol. It is also a cardio-/neuro-/pulmonary toxin that causes reduced human-lung capacity, bradycardia, and heart arrhythmias after only 15-second, 16,000 µg/m^3^ airborne exposures; at higher doses, TCFM causes cognitive malfunction, pneumonia, lung inflammation, hemorrhage, pleuritis, and pericarditis [52]. Yet this dangerous, 15-second, 16,000 µg/m^3^ TCFM dose is lower than 86% of Canoga Parks’ soil-gas levels that could enter indoor air [41]. CBRE/TCC also errs in claiming that TCFM is not a carcinogen; because TCFM has been banned for 34 years, it has not had carcinogenicity evaluation, though prior to the ban, workers experienced the previously mentioned acute effects [53].

*Second*, CBRE/TCC’s 2018 below-all-screening-levels claim is false because when the Canoga Park sampling was conducted in 2018, the 2018 EPA TCFM-screening/health-protective level was 730 µg/m^3^ [53]. Yet this is a TCFM level that 100% of Canoga Park soil-gas samples violate; indeed, 80% of Canoga Park soil-gas samples violate this 730 µg/m^3^ level by three orders of magnitude [43]. Moreover, CBRE/TCC used a 2017 screening/health-protective level at other sites of 1300 µg/m^3^ TCFM [39], and 93% of all Canoga Park soil-gas samples violate CBRE/TCC’s own protective level; 80% of all Canoga Park soil-gas samples violate this health-protective level by at least two orders of magnitude [43].

*Finally*, CBRE/TCC’s 2018 claim that contaminants exhibit no increases with depth is false because when the Canoga Park sampling was conducted, all three soil-gas-sampling locations showed increasing-with-depth TCFM [41] (Table 1, Figure 4). Sampling showed increases from 8900 to 140,000 µg/m^3^ (6 to 47 ft); from 170,000 to 260,000 µg/m^3^ (5.6 to 33 ft); and from 140,000 to 170,000 µg/m^3^ (6 to 35.5 ft). Respectively, these Canoga Park samples show 1600%, 150%, and 120% TCFM increases with depth [41]—yet another way in CBRE/TC data contradict CBRE/TCC’s claims to the regulator and the public. 

#### 4.1.2. Sampling Misrepresentations/Non-Representativeness Threaten Environmental Justice and Equality

The preceding examples of misrepresentations, non-representative sampling, and health-threatening pollution underestimates will disproportionately affect all four toxic-site neighborhoods, as subsequent sections show that all four sites are environmental-justice communities. The failure to adequately assess/sample toxic-site contaminants, as shown by our PDQA results, exacerbates already-existing, inequitable pollution and health harm.

As subsequent paragraphs show, all four sites are environmental-justice communities, home to disproportionate numbers of children, Hispanics, and poor people (see the authors’ Table 9). For instance, as already mentioned, the Pasadena and Monrovia toxic-site, apartment-residence redevelopments are both located beside 10-lane Interstate 210, a main Los Angeles diesel-truck artery. Yet Pasadena’s beside-Interstate-210 census blocks house the poorest two quintiles of California residents [54], disproportionate concentrations of Hispanics [55], and disproportionate concentrations of children, as California-Hispanic households average 88% more children than California households generally [56]. Other Pasadena-toxic-site, sensitive populations include patients at the hospital-size, Kaiser Permanente medical/urgent-care facility abutting the east side of the toxic site, and the middle-/high-school/college students taking courses at the community college abutting half of the north side of the toxic site.

Monrovia’s beside-Interstate-210 census blocks likewise house the poorest two quintiles of California residents [57]. These census blocks also are home to disproportionate concentrations of Hispanics [58], and 88% (see preceding paragraph) more children than California generally [56] (see Table 9).

In the Canoga Park neighborhood of Los Angeles, poverty is 85% higher [59] and 44% more households receive food stamps [60,61] than California generally. Likewise, the percentage of Hispanics is 62% higher [62], and their households include 88% more children than California generally [56].

The Boyle Heights neighborhood of Los Angeles has a poverty rate 130% higher than California generally, its Hispanic population is 147% higher than California generally, and its rate of households with age-18-and-under children is 42% higher than for California as a whole [63]. Boyle Heights is also among the state’s 4% worst-polluted census tracts [64]. Hence, apart from the flawed and non-representative toxic-site testing shown in the preceding results, all four neighborhoods—where the toxic sites are located—house disproportionate numbers of children, poor people, and Latinos, all environmental-justice populations who are repeatedly subjected to pollution inequalities (see the authors’ Table 9). Hence the non-representative sampling could exacerbate these already-existing inequalities. 

### 4.2. Limitations and Future Directions

Regarding *limitations* of this analysis, this article provides only a *preliminary* DQA, as it covers only soil-gas sampling at only four semi-privatized urban-infill-development, VOC-contaminated sites. Our preliminary assessment also identifies only DQA representative-sampling violations. As such, it provides neither a full DQA nor any quantitative evaluation of health harm resulting from site-risk underestimates and failure to follow government soil-gas sampling “requirements” that are needed to ensure data quality [17].

*Future research* might examine non-urban-infill, non-semi-privatized, non-California, non-US, non-CBRE/TCC assessments/cleanups, including those with non-VOC contaminants, to see whether future results are consistent with our results. Researchers also might examine exposures/potential health risks associated with “remediated” toxic sites, including any association between DQA, representativeness, or other sampling violations and resulting health harm, environmental injustice, and environmental-health inequality.

Regarding *future policy*, if our results can be replicated, it might be desirable to add representative-sampling/other technical, scientific requirements, including third-party testing/remediation oversight, to the implementation and lender documents mandated by the UN’s New Urban Agenda [8]. Future public-health policies might also warn of potential threats from remediated sites and investigate additional ways to prevent both non-representative sampling at toxic sites and resulting health threats caused by nonrepresentative sampling.

## 5. Conclusions

Our results confirm our hypothesis that interested parties’/redevelopers’ semi-privatized testing of urban-infill, hazardous-waste sites contaminated with volatile organic compounds may be non-representative, fail data-quality analysis, fail to adequately assess carcinogenic vapor intrusion into buildings, and therefore fail to address pollution threats and environmental injustice/inequality. We tested our hypothesis by determining whether sampling/assessments conducted by the world’s largest commercial developer, CBRE/TCC, met government-mandated “requirements” for volatile-organic-compound sampling. All but one of the CBRE/TCC toxic-site redevelopments assessed in this study violated all 10 of the government-mandated “requirements” for ensuring the temporal, geographical, and technological representativeness of hazardous-site sampling. The one remaining CBRE/TCC toxic site violated 9 of the 10 government-mandated sampling requirements. 

Assessing the consequences of the preceding 39 of 40 possible violations of government requirements for scientific sampling, our discussion showed that these violations uniformly cause health-risk underestimation. As a result, these violations cause at least four distinct public-health threats to children, minorities, and poor people, as well as a worsening of environmental inequities. That is, all the CBRE/TCC toxic-site redevelopments are located in environmental-justice communities, neighborhoods with disproportionate numbers of children, minorities, and poor people.

If our results can be replicated at other toxic sites, then society may need to devise and require new safeguards in order to ensure protective toxic-site redevelopment. In particular, society may need to re-examine how to ensure that semi-privatized testing and cleanup serves not mainly private profits but also public health, environmental justice, and environmental-health equity.

## Figures and Tables

**Table 1:** Method 1 criteria for selecting which toxic-site assessments to evaluate.

Criteria	Requirements
**1**	Toxic-site-urban-infill redevelopment
**2**	Testing/remediation through partly privatized schemes
**3**	Main site toxins are carcinogenic/genotoxic VOCs
**4**	Testing/remediation/redevelopment by CBRE/TCC
**5**	Location in California
**6**	Soil-gas testing has been conducted
**7**	Site assessments prepared since 2011
**8**	Accessible site documents and soil-gas-testing data logs
**9**	Contamination with carcinogenic/genotoxic TCE

**Table 2:** Representativeness questions based on US Environmental Protection Agency (EPA) [18] and California (CA) Department of Toxic Substances Control (DTSC) [17] testing guidance.

Category	Question Number	Representativeness Question [17]
*Temporal* *representativeness* [18]		
	RepQ1	Are samples less than 3 years, preferably within 1 year, old?
*Geographical* *representativeness* [18]		
	RepQ2	Were data collected near contaminant sources?
	RepQ3	Do samples trace the full extent of vapor-phase-contaminant plumes?
	RepQ4	Do samples provide a 3-D delineation of all subsurface contaminants?
	RepQ5	Are data dense enough to provide isoconcentration contours for all toxins?
	RepQ6	Do data provide at least two sub-slab sampling locations per building?
*Technological* *representativeness* [18]		
	RepQ7	Are method-reporting limits sensitive enough for a “screening evaluation”?
	RepQ8	Were samples from permanent/semi-permanent soil-gas wells?
	RepQ9	Did soil-gas-sampling output show seasonal/temporal variations?
	RepQ10	Were soil-gas samples taken under “steady-state conditions”?

**Table 3:** Temporal representativeness of soil-gas sampling (RepQ1) at four toxic sites in California.

	Pasadena [39]	Monrovia [40]	Canoga Park [41]	Boyle Heights [43]
Year of sampling	2007	2011	2012	2017
Year data submitted	2017	2020	2017	2019
Years between sampling and data submission that US EPA says is necessary to “guarantee temporal representativeness” [18]	1	1	1	1
Upper limit of years between sampling and data submission that “must” not be violated, according to US EPA [18]	3	3	3	3
Years between sampling and data submission, this site	10	9	5	2

**Table 4:** Examples of CBRE/TCC human-carcinogen detection/reporting limits (RepQ7), 2017–2020, that are required to be as sensitive as California DTSC screening levels.

	2017 Pasadena	2020 Monrovia	2017 Canoga Par	2018 Boyle Heights
DTSC health-protective/screening levels, taken from CBRE/TCC 2017 assessment [39].	0.067 µg/m^3^	0.48 µg/m^3^	0.46 µg/m^3^	0.48 µg/m^3^
CBRE/TCC 2017 detection/reporting limit used at each site	20 µg/m^3^ [39]	20 µg/m^3^ [40]	3.4 µg/m^3^ [41]	100 µg/m^3^ [43]
Contaminant example	Carbon tetrachloride	Trichloroethylene	Perchloroethylene	Trichloroethylene
Contaminant is a no-safe-dose genotoxin	No	Yes	Possibly	Yes

**Table 5:** Examples of RepQ8–9 in CBRE/TCC soil-gas assessments 2017–2020.

	2017 Pasadena US Navy Weapons Site [39]	2020 Monrovia Industrial Site [40]	2017 Canoga Park Nuclear-Missile Site [41]	2018 Boyle Heights Railroad Site [43]
Toxic-site acres	9	10	51	18
Semi-permanent/permanent wells	0	0	0	5% (15 wells)
Wells capturing seasonal/temporal contaminant fluctuations	0	0	0	5%
Most soil-gas wells have higher contaminant levels, last 2 years	No sampling, last 2 years	No sampling, last 2 years	No sampling, last 2 years	Yes

**Table 6:** Examples of steady-state contamination (RepQ10) in CBRE/TCC soil-gas assessments, 2017–2020.

	2017 Pasadena US Navy Weapons Site [39]	2020 Monrovia Industrial Site [40]	2017 Canoga Park Nuclear-Missile Site [41]	2018 Boyle Heights Railroad Site [43]
Most wells have increasing-with-depth contaminants	Yes	0 same-location, multi-depth samples	Yes	Yes
Most wells have higher contaminant levels, last 2 years	No sampling, last 2 years	No sampling, last 2 years	No sampling, last 2 years	Yes
Confirmation of steady-state contaminant conditions, thus non-migration of contaminants	No	No	No	No
Possible carcinogenic vapor intrusion into onsite buildings	Yes	Yes	Yes	Yes

**Table 7:** Redevelopment toxic sites with trichloroethylene (TCE) and other volatile-organic-compound (VOC) contaminants.

CA CBRE/TCC Toxic Site	Former US Military, Nuclear and Missile Development, Testing, and Production Site?	Residential Development?	Sitewide Soil-Gas Survey Conducted?
Pasadena [39,44]	Yes	Yes	No
Monrovia [40]	No; industrial	Yes	No
Canoga Park [41,42]	Yes	No; commercial	No
Boyle Heights [43]	No; industrial	No; commercial	No

**Table 8:** CBRE/TCC misrepresentations of the quality of its toxic-waste sampling.

CA CBRE/TCC Toxic Site	Is There Randomized or Grid-Based Sampling, Needed for Waste and Risk Characterization [45]?	Does Sampling Reveal Extent of Toxic Waste?	Does Sampling Reveal Magnitude of Toxic Waste?	Did CBRE/TCC Claim All Site Contaminants “Were Fully Investigated” [50] or Addressed [43]?
Pasadena	No [44]	No, given violations of RepQ3–4 and RepQ5, 8–9 [39,44]	No, given violations of RepQ2,6 and RepQ5, 8–9 [39,44]	Yes [50]
Monrovia	No [40]	No, given violations of RepQ3–4 and RepQ5, 8–9 [40]	No, given violations of RepQ2,6 and RepQ5, 8–9 [40]	Yes [51]
Canoga Park	No [41,42]	No, given violations of RepQ3–4 and RepQ5, 8–9 [41,42]	No, given violations of RepQ2,6 and RepQ5,8–9 [41,42]	Yes [41]
Boyle Heights	No [43]	No, given violations of RepQ3–4 and RepQ5,8–9 [43]	No, given violations of RepQ2,6 and RepQ5, 8–9 [43]	Yes [43]

**Table 9:** Toxic-site demographic and environmental-justice characteristics.

Neighborhood of CA Toxic Site	Poverty Rate	Latino Population	Child Population
City of Pasadena, census tracts that abut freeway, as toxic site does	Poorest two quintiles in California [54]	24% higher than CA average [55]	Up to 88% higher than average CA households [46,56]
City of Monrovia, census tracts that abut freeway, as toxic site does	Poorest two quintiles in California [57]	13% higher than CA average [58]	Up to 88% higher than average CA households [46,56]
Canoga Park neighborhood of Los Angeles	85% higher than CA average [59]	62% higher than CA average [62]	Up to 88% higher than average CA households [56]
Boyle Heights neighborhood of Los Angeles	130% higher than CA average [63]	147% higher than CA average [63]	42% higher than average CA households [63]

## Data Availability

No new data were created or analyzed in this study. Data sharing is not applicable to this article. All data supporting reported results can be found online, in the respective citations listed in this paper.

## Abbreviations: Acronyms Used in the Article Acronym Phrase

**Acronym**	**Phrase**
ATSDR	US Agency for Toxic Substances Disease Registry
CARB	California Air Resources Board
CERCLA	Comprehensive Environmental Response, Compensation, and Liability Act
CSOR	California Senate Office of Research
DQA	data-quality-assessment
DTSC	California Department of Toxic Substances Control
EASI	Environmental Asset Services, Inc.
EC	European Commission
EPA	US Environmental Protection Agency
EU	European Union
NOTSPA	US Naval Ordnance Testing Station, Pasadena, California
OEHHA	California Office of Environmental Health Hazard Assessment
PCE	perchloroethylene (tetrachloroethylene)
PDQA	preliminary data-quality assessment
PE-GCS	Pure Earth and Green Cross Switzerland
RCC	Retail Compliance Center
RepQ	representativeness questions
TCC	Trammell Crow Company
TCE	trichloroethylene
TCFM	trichlorofluoromethane
UN	United Nations
UNGA	UN General Assembly
US	United States
**VI**	**vapor intrusion**
VOC	volatile organic compound
WB	World Bank Group
WHO	World Health Organization
WoE	weight-of-evidence
