# Correction: Shrader-Frechette, K.; Biondo, A.M. Data-Quality Assessment Signals Toxic-Site Safety Threats and Environmental Injustices. *Int. J. Environ. Res. Public Health* 2021, *18*, 2012

**DOI:** 10.3390/ijerph181910293

**Published:** 2021-09-29

**Authors:** Kristin Shrader-Frechette, Andrew M. Biondo

**Affiliations:** 1 Department of Biological Sciences, 100 Malloy Hall, University of Notre Dame, Notre Dame, IN 46556, USA; 2 Department of Economics, 3060 Jenkins Nanovic Hall, University of Notre Dame, Notre Dame, IN 46556, USA; abiondo@nd.edu

The authors would like to make the following corrections (1 and 2) to this paper [1].

Regarding correction 1, in the earlier paper we inadvertently used the statistics for no sampling of currently occupied site buildings instead of current site buildings. Regarding correction 2, we made a typographical error in an earlier draft, exchanging references [41] and [43]. This caused us to misplace data from reference [41] for data from reference [43] in the earlier paper.

1. As a result, part of line 6 of the first paragraph on p. 8 was published as:

“collected no sub-slab samples for 89% of 26 currently occupied buildings”

but, instead, the preceding material should be corrected to:

“collected no required sub-slab samples for 86% of 29 current buildings”.

2. As a result, the third paragraph of p. 12 was published as:

“*Second*, CBRE/TCC’s 2018 below-all-screening-levels claim is false because when the Canoga Park sampling was conducted in 2018, the 2018 EPA TCFM-screening/health-protective level was 730 µg/m^3^ [53]. Yet this is a TCFM level that 100% of Canoga Park soil-gas samples violate; indeed, 80% of Canoga Park soil-gas samples violate this 730 µg/m^3^ level by three orders of magnitude [43]. Moreover, CBRE/TCC used a 2017 screening/health-protective level at other sites of 1300 µg/m^3^ TCFM [39], and 93% of all Canoga Park soil-gas samples violate CBRE/TCC’s own protective level; 80% of all Canoga Park soil-gas samples violate this health-protective level by at least two orders of magnitude [43].”

Instead, the preceding paragraph should be corrected to:

“*Second*, CBRE/TCC’s 2018 below-all-screening-levels claim is false because, when the Canoga Park sampling was conducted, the ATSDR/EPA TCFM-screening/health-protective soil–gas/indoor-air levels, respectively, were 7300/730 µg/m^3^ [53]. Yet, 92% of Canoga Park soil–gas samples violated this ATSDR/EPA level, and 77% violated this level by two orders of magnitude [41]. Moreover, CBRE/TCC used a 2017 screening/health-protective level at other sites of 1300 µg/m^3^ TCFM [39], and 92% of all Canoga Park soil–gas samples violated CBRE/TCC’s own protective level; 77% of all Canoga Park soil-gas samples violated this health-protective level by at least two orders of magnitude [41].”

The authors would like to apologize for any inconvenience to the readers caused by these errors.
